# Facial Reanimation Surgery: An Investigation on the Role of Online Information Sharing in Patient Education and Decision Making

**DOI:** 10.1177/22925503251322525

**Published:** 2025-04-15

**Authors:** Tiffany T. Ni, Amy Patricia Ruth Graham, Syena Moltaji, Heather L. Baltzer

**Affiliations:** 1 12366 Temerty Faculty of Medicine, University of Toronto, Toronto, ON, Canada; 2 Division of Plastic, Reconstructive, and Aesthetic Surgery, Department of Surgery, 12366 University of Toronto, Toronto, ON, Canada

**Keywords:** facial paralysis, Bell's palsy, facial reanimation, social media, patient education, paralysie faciale, paralysie de Bell, réanimation faciale, réseaux sociaux, l'éducation des patients

## Abstract

**Purpose:** The emergence of facial reanimation surgery as a reconstructive option has sparked a growing interest among patients with facial paralysis, leading to an increase in patients seeking and sharing information on these surgical modalities. This study evaluated the role of social media in information-sharing on facial reanimation surgery. **Methods:** We identified 630 Facebook groups based on the initial keyword search for “facial paralysis” and “Bell's palsy.” Groups with < 100 members, non-English content, or restricted access were excluded. Within each group, searches were conducted for terms related to surgery and posts were categorized as sharing information, seeking information, sharing support, seeking support, or sharing appreciation. **Results:** The search yielded 630 groups; 21 groups met the inclusion criteria (average size = 4037, largest = 31 400). Facial reanimation surgery was discussed in 15 groups, with 487 relevant posts tabulated. In the sharing information axis, posts were related to personal experiences (63%), alternatives (14%), link shares (7%), surgeon/center (5%), general recovery progression (8%), objective information on surgical modality (1%), objective information on nerve injury (1%), and general information on relevant medical research (1%). In the seeking information axis, posts were related to personal experience (71%), objective information (12%), surgeon/center (4%), second opinion (13%), and alternatives (1%). **Conclusion:** Social media is an essential source of information and support for people with facial paralysis. These study findings will inform the implementation of future knowledge translation efforts to maximize education and subsequent uptake of facial reanimation reconstructive surgery.

## Résumé

**Contexte :** L’émergence de la chirurgie de réanimation faciale comme option de reconstruction a déclenché un intérêt croissant chez les patients atteints de paralysie faciale, conduisant un nombre croissant d’entre eux à rechercher et à partager de l’information sur ces modalités chirurgicales. L’étude a évalué le rôle des réseaux sociaux sur le partage d’information sur la chirurgie de réanimation faciale. **Méthodes :** Nous avons identifié 630 groupes Facebook sur la recherche initiale avec les mots-clés « paralysie faciale » et « paralysie de Bell ». Les groupes comptant moins de 100 membres, au contenu en langue non anglaise ou à accès restreint ont été exclus de la sélection. Des recherches ont été menées dans chaque groupe pour des termes liés à la chirurgie et les publications ont été classées selon qu’elles partageaient de l’information ou en recherchaient, partageaient un soutien ou en recherchaient, ou partageaient une appréciation. **Résultats :** La recherche a identifié 630 groupes; 21 groupes satisfaisaient les critères d’inclusion (taille moyenne : 4 037, plus grand groupe : 31 400). La chirurgie de réanimation faciale était discutée dans 15 groupes et 487 messages pertinents ont été mis en tableau. Sur l’axe du partage d’informations, les publications avaient trait aux expériences personnelles (63%), aux options de remplacement (14%), aux partages de liens (7%), à un chirurgien ou centre (5%), à l’évolution générale de la récupération (8%), à une information objective sur la modalité opératoire (1%), à une information objective sur les lésions nerveuses (1%) et à l’information générale sur la recherche médicale pertinente (1%). Sur l’axe de la recherche d’informations, les publications avaient trait à une expérience personnelle (71%), une information objective (12%), au chirurgien ou au centre (4%), à une deuxième opinion (13%) et aux options de remplacement (1%). **Conclusion :** Les réseaux sociaux sont une source essentielle d’information et de soutien pour les personnes atteintes de paralysie faciale. Les constatations de cette étude renseigneront la mise en œuvre des efforts de traduction des connaissances futures pour accroître au maximum l’éducation des patients et le développement subséquent de la chirurgie reconstructrice de réanimation faciale.

## Background

Facial animation plays an integral role in human communication, allowing us to express emotions and provide nonverbal cues. Most commonly, the cause of facial nerve palsy remains unknown and is termed “Bell's Palsy.”^1^ Facial paralysis can have devastating outcomes on one's quality of life. Previous studies have found that patients with long-term facial nerve paralysis were reported to have lower social functioning, higher rates of anxiety and depression, and physical complications relating to paralysis, such as diminished oral continence and poor speech.^2–4^

Facial paralysis management includes pharmacological therapy, physical therapy, and both dynamic and static surgical interventions.^5,6^ Effective surgical outcomes depend on the time between paralysis onset and surgery.^1,5,7^ Delays can lead to irreversible muscle atrophy, making procedures like cross-face nerve grafts (CFNG) or masseter nerve transfers ineffective due to the lack of muscle targets.^8^ Timely treatment is crucial for better outcomes, highlighting the need for patients to be informed about current surgical options.^9^ This knowledge gap may especially impact patients in rural or remote areas with limited access to specialists.^10,11^

To address this knowledge gap, it is essential to understand where patients seek information on treatment options and the types of information available. Rudy et al found growing public interest in facial paralysis and reanimation surgery on Twitter, driven by increased surgeon publications and discussions.^12^ However, their study did not explore social media as a support and information source for patients actively seeking treatment. With over 2.5 billion Facebook users and numerous groups on this condition, Facebook has become a key platform for patients and caregivers.^12–15^

The purpose of this current study is to assess the use of Facebook as a social media platform in information-sharing within the facial paralysis community, specifically to review the discussion of facial reanimation surgeries to better understand online communities’ role in medical education, patient perspectives, and patient decision-making.

## Methods

### Search Strategy

Publicly available data was collected from the online social media network, Facebook, from August 1 to 31, 2022. The group function of Facebook was searched with the key terms “Bell's Palsy” and “Facial paralysis.”

Both public and private Facebook groups pertaining to facial paralysis were searched. To access private groups, a standardized statement of intent was sent, outlining the study purpose, a declaration stating that no posts would be made from this account and that the groups would be accessed for 24 h. While some groups explicitly limited membership to individuals living with facial paralysis, the study authors made attempts to join all such groups, providing a clear explanation of our research purpose and its potential benefits to the community. Groups were excluded if the formal request to join was explicitly denied by group administrators or if no response was received after a 14-day waiting period. The specific breakdown of exclusion reasons within this category, (1) explicit rules limiting membership to individuals living with facial paralysis and (2) lack of response by group administrators, was not explicitly tracked.

In addition, Facebook groups were excluded if they had < 100 members, and the group access was limited to individuals living with facial paralysis, containing contents not related to facial paralysis or in a non-English language. Groups with < 100 members were excluded to minimize overlapping membership with larger, more established groups and to ensure the analysis focused on capturing the most active and substantial discussions within the larger online communities related to Bell's palsy and facial paralysis.

Data collected on each group page included the group name and number of members.

Each included group was then searched for the terms “surgery,” “decompression,” “repair,” “graft,” “transfer,” and “operation” to identify relevant posts on facial reanimation surgery, including (1) relevant posts about facial reanimation surgery, (2) relevant comments on posts about facial reanimation surgery, and (3) relevant comments on posts not about facial reanimation surgery. The respective posts were then tabulated and analyzed. No personal identifiers of group members or postauthors were recorded. In all analyzed comments or post content, pseudonyms were utilized to maintain confidentiality, and locations were altered to ensure the anonymity and privacy of the participants.

### Data Analysis

Extracted data was categorized into the following 5 following axes for further analysis:

1. Seeking information: This category includes posts where individuals request knowledge or advice regarding facial reanimation surgery. The inquiries within this axis vary in scope, from personal anecdotes of surgical outcomes to more objective requests for clinical information. Commonly, users ask for descriptions of others’ experiences with specific surgical procedures, such as cross-face nerve grafts or muscle transfers, in an attempt to understand potential risks, benefits, and recovery timelines. Additionally, posts may include requests for recommendations on surgeons or medical centers that specialize in these procedures, as well as queries about obtaining second opinions on surgical options or treatment plans. This axis highlights the informational needs of patients who are navigating their treatment options.
2. Sharing information: Posts in this axis involve individuals providing information to the community. This includes personal stories of undergoing facial reanimation surgery, along with sharing objective data such as the details of their procedures, outcomes, and recovery processes. Users may also contribute alternative treatment methods, including nonsurgical options, and share links to external resources such as medical literature or patient education materials. Additionally, posts recommending specific surgeons or medical centers based on personal experience are common. This axis illustrates how patients leverage personal narratives to educate others, supplementing clinical knowledge with personal experiential insights.
3. Sharing support: Posts within the sharing support axis are centered on emotional and moral support. These posts often involve users offering words of encouragement, reassurance, and solidarity to others who are dealing with facial paralysis or considering surgical intervention. Members may express empathy, provide comfort during difficult times, or offer condolences to those facing complications or setbacks. This axis underscores the community's role in providing psychological and emotional reinforcement, fostering a sense of belonging and understanding.
4. Sharing appreciation: Posts categorized in this axis demonstrate gratitude toward other group members or the community as a whole, expressing thank those who have shared their experiences, offered advice, or provided support. Posts in this axis reflect appreciation for helpful information or emotional support, with users acknowledging the value of the collective knowledge and empathy offered within the group. This axis highlights the reciprocal nature of online community engagement, where users not only seek or provide help but also express gratitude for the contributions of others.
5. Seeking support: This axis comprises posts where individuals are actively seeking emotional or empathetic assistance from others in the group. These posts come from individuals who are experiencing emotional distress, uncertainty, or fear related to their facial paralysis and/or upcoming surgery. Further, the posts are focused on obtaining emotional reassurance, encouragement, and understanding from peers who have undergone similar experiences. This category illustrates the reliance on peer-to-peer emotional support that is prevalent within patient-led online communities.

## Results

**Figure 1.**
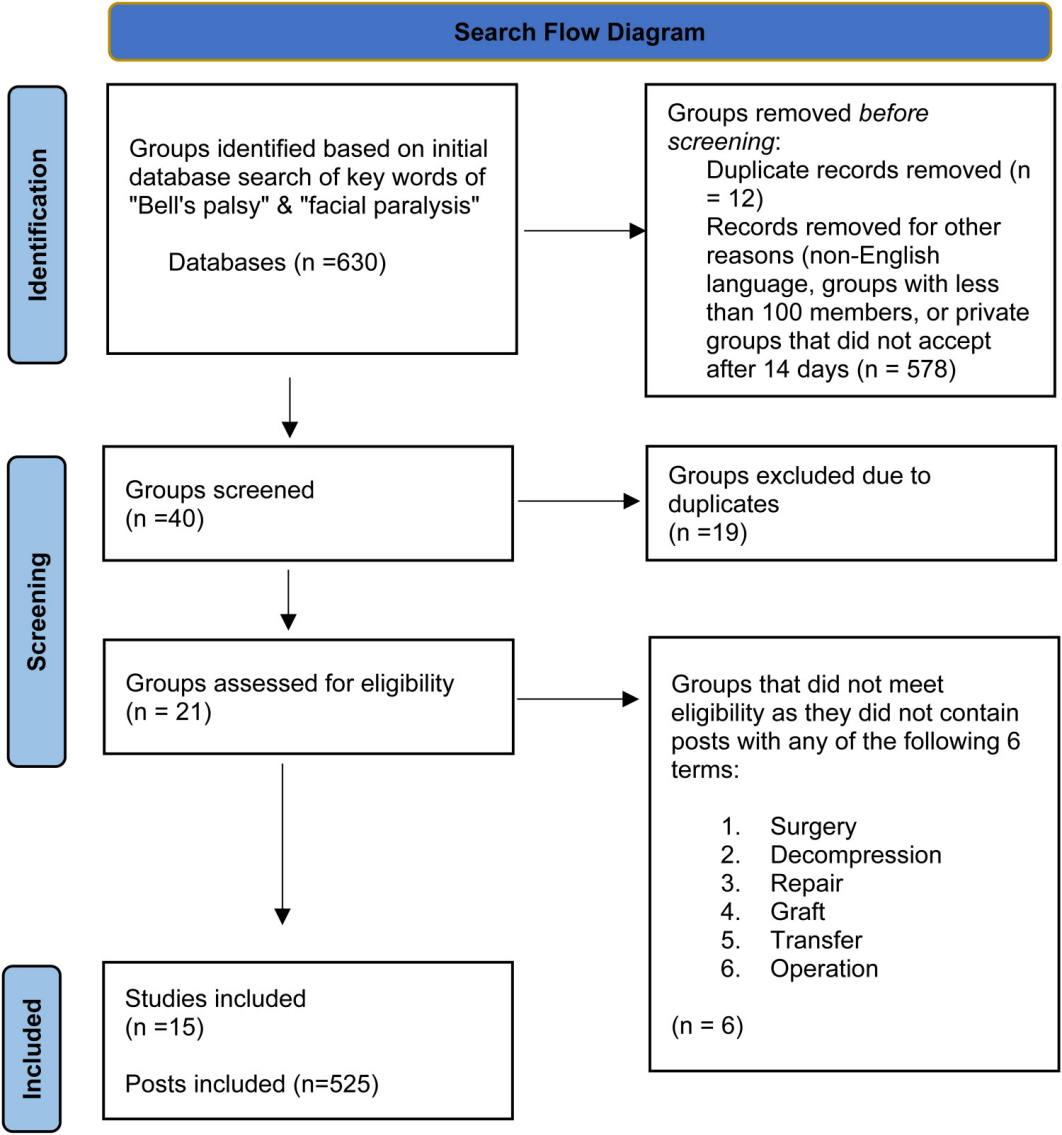
Search flow diagram.

Our search yielded a total of 630 Facebook groups, of which 21 were included in this study, and 609 were excluded (Figure 1). The average membership number of included groups was 15 562 members (largest group = 31k members, smallest group = 125 members). Facial reanimation surgery was discussed in 15 groups, with 525 total posts on facial reanimation surgery across all 15 groups (n = 252 seeking information, 158 = sharing information, n = 73 seeking support, n = 28 sharing support, and n=14 sharing appreciation). Of the 15 groups assessed for mention of facial reanimation surgery, 11 were private, and 4 were public.

The initial search with “Bell's palsy” and “facial paralysis” returned 630 Facebook groups. After applying the inclusion and exclusion criteria, 40 groups were selected for further assessment. Due to the overlap between the keywords, 19 groups appeared in both searches, highlighting the interchangeable use of terms in online discussions. Excluding this overlap, 21 unique groups were further evaluated for the 6 specific search terms related to facial reanimation surgery. Of these 21 groups, 15 groups contained substantive discussions pertinent to facial reanimation surgery and were thus included in the final analysis, contributing to the 525 total posts examined in this study. This rigorous selection process ensured that the analysis captured a diverse online discourse relevant to facial reanimation surgery while minimizing data duplication.

Due to space limitations, full quotes were not included in the tables and figures. However, all 525 posts in the final analysis met at least one of the 6 predefined search criteria and contained either “Bell's palsy” or “facial paralysis” as per our methodology. The excerpts in the figures were selected to capture key points while maintaining clarity and conciseness. This approach follows qualitative research practices, where representative quotes illustrate key themes and patterns in the data.^16^

A total of 158 posts were categorized into the “sharing information” axis, with the highest number of posts (63%) relating to personal experiences, followed by alternatives (14%), link shares (7%), surgeon/center information (5%), general recovery progression (8%), objective information on surgical modality (1%), objective information on nerve injury (1%), and general information on relevant medical research (1%). Representative data points from this axis are presented in Table 1.

**Table 1.** Sharing Information Axis: Description, Prevalence, and Paraphrased Representative Quotes by Theme.

Sharing information axis	Axis description	Prevalence, n (%)	Representative quotes
Surgical intervention update	Individual narrative relaying personal experience of a facial reanimation procedure	99 (63%)	“Hi everyone, it is 5 days after my surgery cross graft, anastomosis. My face is still swollen like a lot, but doctor said it will be okey by time. You can't see it because bandage. My leg doesn't hurt, I was walking normally day after surgery. They took nerve from the leg, that nerve is for sensation, so it is little bit weird now in my leg. Doctors said they are happy how the surgery went. Now, for 3 months I need to rest and after that I will start PT and wait for results. I like my new tattoos. Let's wake up my smile back.”
Alternatives	Sharing information on treatments to restore facial function outside of surgical modalities	22 (14%)	“(Botox) ok you guys so I finally gave in and got Botox on Friday 24 Units 9 Shots—at the Plastic Surgery Unit at [*redacted*] The Dr told me this appointment is ONLY to get Botox No Questions ‘I’m Only doing Botox to help with Symmetry’! I was fine all day until the evening I felt really sleepy and crashed on my couch.”
Link share	Sharing links to external sites with information on facial reanimation procedures (ie, news outlets and resources)	11 (7%)	“BP in the news: [Link to Daily Mail's article: Paralysed man who hadn't smiled in years will grin again thanks to breakthrough nerve ‘transfer’ surgery].”
Surgeon/Center	Providing the name of a surgeon performing facial reanimation procedures or a center where the procedure is performed	8 (5%)	“TOP DOCS: Seems like the top docs that specialize in facial paralysis are Dr [*redacted*], Plastic Surgeon in Cali and Dr [*redacted*]. Otolaryngologist in Boston, Massachusetts.”
General progression of recovery	Sharing individual narrative of first-hand experience of recovery without indication of the modality of the intervention	13 (8%)	“Mine is 2nd degree.”
Objective information on surgical modality	Sharing information on facial reanimation surgery, investigations, surgical candidacy, and rehabilitative process	2 (1%)	“Nerve transfer—connecting the masseter nerve to facial muscle.”
Objective information on nerve injury	Sharing information on nerve injury, investigations, surgical candidacy, and rehabilitative process	2 (1%)	“The 3 degrees of facial nerve injury 1^st^ degree injury—The 1^st^ degree injury is where the nerve is irritated enough that it stops firing, but everything is intact…”
General information on research related to Bell's palsy	Sharing general information on research related to Bell's palsy (ie, not exclusively surgically related)	1 (0.6%)	“I don't understand all this, but the last sentence caught my eye.” “There is unlikely to be further research into the role of an operation because Bell's palsy usually recovers without treatment.”

Of the 252 posts analyzed within the “seeking information” category, the majority were related to seeking personal experiences (71%), followed by objective information (12%), second opinion seeking (13%), surgeon/center information (4%), and alternatives (1%). Representative data points from this axis are presented in Table 2.

**Table 2.** Seeking Information Axis: Description, Prevalence, and Paraphrased Representative Quotes by Theme.

Seeking information axis	Axis description	Prevalence, *n* (%)	Representative quotes
Personal experience	Seeking individual narrative of first-hand experience with facial reanimation procedures	178 (71%)	“Did anyone go through a surgery to fix your smile and the nerve damage from Bell's palsy? I have a few questions. I am going for a surgery next week and I’m very nervous going in. I’ve had BP for 5 years now and it hasn't fully recovered. Any information or guidance would be great.”
Objective information	Seeking information on facial reanimation surgery, investigations, surgical candidacy, and rehabilitative process	29 (12%)	“Is it good to wait for years and not do surgery? I heard surgery like facial reanimation or cross face nerve graft helps a lot with Bell palsy if no improvement after 6–12 months. Any thoughts”?
Surgeon/center	Requesting contacts for a qualified surgeon or a specific hospital where facial reanimation procedures are performed	11 (4%)	“Hey, has anyone here had surgery for facial paralysis. And what kind of surgery? I am curious about your experiences and results. I live in the Netherlands so I'm looking for a good surgeon, preferably in Europe. So, if you know a good doctor please let me know.”
Second opinion	Seeking further information or alternative perspectives regarding facial reanimation surgery after an initial consult or physician's orders	32 (13%)	“Hoping to get some thoughts on this. I was due to have weight loss surgery on the 17^th^ Jan. however went with BP in the 3^rd^ of Jan instead. So no real recovery yet for BP, doing physio. My surgeon has rescheduled for 14 Feb. Just thoughts on this please how it might effect my BP. Has anyone had any surgery with in a few months of getting BP. Thanks.”
Alternatives	Seeking information on treatments to restore facial function outside of surgical modalities	2 (1%)	“Has anyone found resources for treatments for Synkinesis that differ from the standard recommendations, that is, solutions outside of the US, trials, etc …”

Within the “sharing support” axis, out of the 28 total posts, the highest proportion (82%) involved personal experiences, followed by alternatives (7%), surgeon/center information (4%), awareness efforts (4%), and direct messages to other group members (4%). Representative data points from this axis are presented in Table 3.

**Table 3.** Sharing Support Axis: Description, Prevalence, and Paraphrased Representative Quotes by Theme.

Sharing support axis	Axis description	Prevalence, *n* (%)	Representative quotes
Alternatives	Sharing support through the treatments to restore facial function outside of surgical modalities.	2 (7%)	“Today is 4 months living with friend Bell Palsy. (tumor surgery at December, vestibular schwannoma, 4days after surgery I got covid in hospital). Still any movement, only thing that its better is closing my eye.I started acupuncture months ago. (2x per week). Physical therapy for 3 months now.*B-complex, nimotop. Hope it will be better. What I learned that's how to look pretty much OK for selfie have a good day! Tomorrow is Easter, lets enjoy.”
Personal experience	Sharing support though an individual narrative relaying experience of a facial reanimation procedure	23 (82%)	“Trust the process. 8 month post selective neurolysis surgery. Just keep swimming. Never be perfect but starting to accept my new normal. I had paralyses 2 years 6 weeks ago. Gave myself a year to recover before seeing a surgeon. I have scars that are barely visible. He did my eyes. Forehead. Selective neurolysis and symmetrical lift.”
Surgeon/Center	Sharing support through information on a qualified surgeon or a specific hospital where facial reanimation procedures are performed	1 (4%)	“…Next week I’m going to fulfill an old dream, I’m going to Baltimore, USA to meet DR [*redacted*] and get surgery. Just wanted to share my story and give some hope to those who still straggling with this unpleasant condition. Thanks.”
Awareness efforts	Disseminating support via awareness strategies	1 (4%)	“This arrived today and I’m so happy. I’m going to take the flyers and posters to my GP surgery and to other local ones too. I will be wearing my T-shirt all week if I can, I will wash it dry it then put it straight back on. Sending lots of healing love to all RHS and BP sufferers.”
Directly to another group member	Support directed toward another post in the group	1 (4%)	“[*redacted*]—Have you considered waiting until he is older so that he can make the decision himself? From what I've read on the Bells Palsy sight, the surgery could make things worse. Talk to people on the BP forum (link in previous post).”

Within the “seeking support” axis, out of the 73 total posts, the majority (86%) were related to personal experiences, while others sought support through connecting with others (6%), alternative approaches (1%), and seeking financial assistance (3%). Representative data points from this axis are presented in Table 4.

**Table 4.** Seeking Support Axis: Description, Prevalence, and Paraphrased Representative Quotes by Theme.

Seeking support axis	Axis description	Prevalence, *n* (%)	Representative quotes
Personal experience, Surgical procedure	Seeking support based on one's personal experience with facial reanimation procedures	64 (86%)	“Day 7 after DAO surgery. Stitches are still in (not dissolved) and swelling has gone down a bit externally, but there's still some internally. Maybe 60% healed from the surgery? Feeling so impatient!.”
Other's personal experience	Seeking support based on another member's personal experience with facial reanimation procedures	5 (6%)	“Hi everyone! It would be nice if all people who had surgery CROSS GRAFT (taking nerve from leg) can share their pictures and experience. How long was recovery, maybe your scar. In April I will have cross graft and later some other surgery in which doctor will connect also chewing nerve to damaged one. #crossgraft #surgery.”
Connect	Expressing an interest in connecting with a specific population	2 (3%)	“Hi Everyone!! My Daughter just turned 4 but when she was 3 months old she had a tumor that grew and ate up her facial nerve near her mastoid behind the ear … Permanent left side facial paralysis—we have had 1 surgery in NYC and 3 in Boston and 2 more planned before kindergarten! I am looking to connect with any moms or dads who have experience facial paralysis with a child. I would love to hear everyone's story, experience and timeline this is something very near and dear to my heart and not something just anyone can relate to.”
Alternative	Seeking support for the treatments to restore facial function outside of surgical modalities.	1 (1%)	“I was diagnosed with Bell palsy on May 3rd today is exactly one month later. I’ve made a lot of progress, but still struggling with inflammation on the effected side of my face. I used all of the advice you shared on this page, getting plenty of rest, vitamins b12, D3, l-lysine, zinc, vitamin C, turmeric, multi, and omegas which I take daily. I eliminated caffeine and only started acupuncture's last week so far I’ve had 3 sessions and my eye is fully closing, I’m just worried that one eye is a little bigger than the other. I do the massages and facial exercises, but I could do them more often than I’ve been doing. I meditate and pray consistently and daily. If you have any advice for the inflammation please share.”
Financial	Seeking financial support	2 (3%)	[facial photograph included] “Click here to support Funding for surgery organized by *[redacted]*. *[redacted]* has to have Vitrectomy Surgery. One trip to Grand Island to have a shot before surgery…”

Additionally, in the “sharing appreciation” axis, the 14 relevant posts expressed appreciation for the surgery itself (71%), the surgeon/center (14%), and the online group (14%). Representative data points from this axis are presented in Table 5.

**Table 5.** Sharing Appreciation Axis: Description, Prevalence, and Paraphrased Representative Quotes by Theme.

Sharing appreciation axis	Axis description	Prevalence, *n* (%)	Representative quotes
Appreciation for surgery	Sharing appreciation for facial reanimation procedures	10 (71%)	“I thank God my surgery went great this morning my situation could be worse I thank God for all my trials I wouldn't be the strong woman that I am I know I’m Blessed because every prayer I pray God hears me I’ve learned to also to take better care of me and love me because the doctors say this can be caused by stress I love you *[redacted]*!!.”
Appreciation for surgeon/center	Sharing appreciation for a specific surgeon or center involves in facial reanimation surgery	2 (14%)	“After 11 long years and failed Botox attempts, I am finally getting surgery in January! Thanks to another member of this group who recommended a great surgeon, one of the best in the country! I am nervous yet excited. Getting DAO resection and platysmyectomy. If this doesn't give me good results, we are going forward with selective neurolysis. I will for sure post before and after photos.”
Appreciation for group	Expressing gratitude for the platform	2 (14%)	“Quick update, I go in for my exploratory surgery in the morning for the blob that shouldn't be in my sinuses that was highlighted by the scan for my Bell's. In case it all goes sideways a quick and heartfelt thanks to all of you on here who have given me the answers that my didn't have and ways of coping with this strange condition.”

## Discussion

Facial reanimation surgery has emerged as a relatively new reconstructive option for facial paralysis. To enhance patient education, providers must understand the current information shared within facial paralysis communities on social media. Our study reveals a significant gap in accurate information about facial reanimation surgeries online. We found that social media content is often variable in accuracy and heavily reliant on anecdotal evidence (63%). For instance, one user shared, “I did the decompression surgery 2 months after my accident, which I believe is a total waste of time because it doesn't fully heal.” Additionally, most users seeking information preferred personal narratives about facial reanimation procedures (71%), with one asking, “What kind of surgery can fix it? Please share [so] I [can] feel the exact same way you do.” This study highlights significant knowledge gaps and underscores the need for accurate information. It also reflects a strong patient desire for reliable insights from others with similar experiences, guiding future clinician efforts to educate the Bell's palsy community on treatment options.

Facial reanimation surgical procedures, such as cross-face nerve graft, can greatly improve facial symmetry at rest and during voluntary and emotional expression.^4^ However, the outcome highly depends on the time between facial paralysis onset to the surgery.^17^ Unfortunately, for patients experiencing delays in accessing care, progressive atrophy of the facial muscles can render this procedure ineffective due to the lack of a muscle target. Given the time-sensitive nature of this intervention, accurate and timely knowledge dissemination of treatment options is crucial for individuals with facial paralysis who wish to improve their facial symmetry.^8,9^ In our review, we noted confusion among patients regarding the recommended time to intervention, with one patient informing to other group members, “My instincts and my God are telling me to wait, so I’m leaning towards that.”

Over the past decades, there has been a shift in how patients access information and connect with others. Social media, particularly private groups, now allows patients to share experiences more freely than relying solely on clinicians.^12^ Research shows that patients with spinal cord injuries often learn about procedures from peers with similar experiences.^14^ However, on platforms like Facebook, there is a high risk of disseminating unsafe or inaccurate information. For instance, Bernardi et al found that while many posts in the International Hernia Collaboration Facebook Group were helpful, evidence-based management was often lacking, and unsafe recommendations frequently went uncontested.^18^

### Study Design and Scope

This study employed a mixed-methods approach combining qualitative thematic analysis with quantitative content categorization to investigate how facial reanimation surgery is discussed within online communities.

All posts were categorized into 5 thematic axes: (1) seeking information, (2) sharing information, (3) seeking support, (4) sharing support, and (5) sharing appreciation. Content was extracted, paraphrased, and anonymized for each post to protect participant privacy.

Thematic analysis was applied to the qualitative data using a coding dictionary developed by 2 independent investigators (TN and AG). Intercoder reliability was ensured through the involvement of a third researcher (SM) to resolve any discrepancies.^19^ Data saturation was achieved through the iterative process of immersion and crystallization, wherein the 3 authors closely examined and reflected on the data to identify key themes and patterns. This iterative process continued until meaningful and well-substantiated patterns and claims emerged from the data and facilitated the identification of significant trends, providing an empirical and narrative understanding of the topic.

### Information Gaps and Education Challenges in Facial Reanimation Surgery on Social Media

Our study revealed that despite the large number of members in Facebook groups (average size = 4037, largest = 31 400), our search yielded relatively little data on facial reanimation surgeries, with a total of 487 relevant posts tabulated across 15 groups, dating between group inception to August 2022. For reference, within the largest group, titled “Bell's Palsy & Facial Paralysis Support (Group Page),” there have been ∼ 722 new posts within one month. This is in keeping with a lack of current literature on the patient education process on facial reanimation surgery and supports the need for more research on this topic. Interestingly, a previous study by Chu et al^20^ found that the existing online resources on facial plastic surgery procedures often exceeded patient literacy. The complexity of existing written resources represents a potential obstacle to online patient education and decision-making.

Personal information was the most frequently sought form within the seeking information axis, with 71% of individuals seeking first-hand experiences from others who have previously undergone facial reanimation procedures. While peer-generated information on social media offers patients emotional support and reassurance and provides guidance regarding available treatment options, the accuracy of the information is often limited and based on anecdotal evidence. A representative example quote is, “is it good to wait for years and not do surgery? I heard surgery like facial reanimation, or cross-face nerve graft helps a lot with Bell palsy if no improvement after 6–12 months. Any thoughts?” Furthermore, questions regarding the length of time to wait before the procedure, a specific type of facial reanimation surgery, and recovery postop were often not accurately answered. As surgical options are highly variable between patients, this information cannot be easily delineated and outlined via social media. Numerous posts also incorrectly emphasized that the CFNG procedure could only be performed by highly specialized plastic surgeons in the country, with commenters inquiring about accommodations and flights out of the country to receive a consultation.

This study highlights the need for increased awareness and standardization of patient education on facial reanimation surgeries and that social media platforms are critical for health resource dissemination. Individuals with facial paralysis prefer information from peers, using relatable languages, experiences, and images. The findings from the current investigation are vital in guiding future knowledge translation efforts to maximize patient education and subsequent timely uptake of facial reanimation surgery to improve facial symmetry. Specifically, strategies that would benefit patients include (1) standardizing education materials on facial palsy and facial reanimation surgeries, (2) providing a centralized list of surgeons performing the procedure, and (3) establishing an in-person peer-to-peer mentorship group so patients may directly connect with other affected individuals.

This study has several limitations. Firstly, it focuses solely on Facebook, while other major platforms like Twitter and YouTube also host active patient communities. Additionally, some Facebook groups were inaccessible due to privacy settings or denied permissions. This limitation, however, may enhance the generalizability of our findings due to potential overlap between groups. This study also focused on acquired facial paralysis in adults, particularly idiopathic facial palsy, and did not include iatrogenic, neurogenic (eg, stroke), or congenital/pediatric cases. Motivations for online discussions may differ between adults and parents of children, meaning our findings may not fully capture the experiences of the pediatric population. Our exclusion criteria aimed to focus on individuals living with facial paralysis, but some groups permitted the inclusion of family members or others who submitted a statement of intent. As a result, there is a possibility that some participants may not have had direct experience with facial paralysis, which could introduce bias in the data. We also excluded non-English groups, missing insights from non-English speaking patients and how language barriers affect their understanding of facial paralysis and related surgeries. Furthermore, we could not verify the professional qualifications of individuals posting in the groups, making it unclear if posts were from licensed healthcare providers, patients, or others. Future research should explore social media platforms that allow verification of post authors to analyze the relationship between author identity and information quality. While this study aimed to describe the information shared and patient engagement within these communities, assessing the accuracy and misinformation of content is an important area for future investigation.^21,22^

## Conclusion

Patients play a critical role in the shared decision-making process by providing critical insight to surgeons about their disease course and their lived experiences, and in turn, providers offer insights into the latest research, medical, and surgical options available. Facial reanimation surgery can greatly improve the quality of life for patients with facial paralysis. Due to the time-sensitive nature of the condition, timely communication of medical and surgical management options is critical for patients in their recovery. Given the utility of Facebook groups in information dissemination among patients living with facial paralysis, it is of the utmost importance to assess the current discussion of facial reanimation surgeries to understand better online communities’ role in medical education, patient perspectives, and patient decision-making. Taken together, these findings will inform future knowledge translation efforts to maximize education and subsequent uptake of facial reanimation surgery to improve functional outcomes in patients with facial paralysis.

Given the findings of this study, it is evident that there is a gap in patient understanding, particularly regarding facial paralysis and available interventions. The goal of this article is not to critique existing resources but to highlight the reality that many patients turn to online peer communities for support and information. These resources are often less standardized than clinical materials, and some institutions may rely on survivor experiences or clinicians to design educational content. This variability in resources can contribute to patients seeking information outside the clinical setting. As clinicians, we have the opportunity to proactively inform our patients about the existence of these online communities and the potential risks and benefits of engaging with them. Empowering our patients through education and enhancing their ability to make informed decisions about where and how they seek patient information is crucial. It is important to help patients navigate these resources, ensuring they are equipped to critically evaluate the information they encounter, and fostering a collaborative approach to their care.

## Supplemental Material

**Figure:** Video 1.
